# Retraction Note: Exopolysaccharides from *Lactobacillus plantarum* NCU116 induce c-Jun dependent Fas/Fasl-mediated apoptosis via TLR2 in mouse intestinal epithelial cancer cells

**DOI:** 10.1038/s41598-026-70078-4

**Published:** 2026-09-07

**Authors:** Xingtao Zhou, Tao Hong, Qiang Yu, Shaoping Nie, Deming Gong, Tao Xiong, Mingyong Xie

**Affiliations:** 1 State Key Laboratory of Food Science and Technology, Nanchang University, 235 Nanjing East Road, Nanchang, 330047 Jiangxi China; 2 New Zealand Institute of Natural Medicine Research, 8 Ha Crescent, Auckland, 2041 New Zealand

Retraction of: *Scientific Reports* https://doi.org/10.1038/s41598-017-14178-2, published online 27 October 2017

The Authors have retracted this Article.

During post-publication overview of the data included in the Article, the Authors identified multiple image overlaps between Figures 4 and 7. They were, however, unable to find the original data underlying those figures, and have therefore decided to retract the paper.

All Authors agree with the retraction and its wording.

